# High-Intensity Exercise and Hippocampal Integrity in Adults With Cannabis Use Disorder: A Randomized Clinical Trial

**DOI:** 10.1001/jamapsychiatry.2025.2319

**Published:** 2025-09-10

**Authors:** Karyn E. Richardson, Chao Suo, Lucy Albertella, Suzan Maleki, James Coxon, Josh Hendrikse, Sam Hughes, Joseph Pitt, Edouard Kayayan, Catherine Brown, Liam Nguyen, Nadia Solowij, Dan I. Lubman, Rebecca Segrave, Murat Yücel

**Affiliations:** 1 School of Psychological Sciences, Monash University, Melbourne, Victoria, Australia; 2 Monash Biomedical Imaging Facility, Monash University, Clayton, Victoria, Australia; 3 QIMR Berghofer, Herston, Queensland, Australia; 4 School of Psychology, University of Wollongong, Wollongong, New South Wales, Australia; 5 Monash Addiction Research Centre and Eastern Health Clinical School, Monash University, Box Hill, Victoria, Australia; 6 Department of Psychiatry, School of Clinical Sciences, Monash University, Clayton, Victoria, Australia; 7 Turning Point, Eastern Health, Richmond, Australia

## Abstract

**Importance:**

Cannabis is the most commonly used illicit drug, with 10% to 30% of regular users developing cannabis use disorder (CUD), a condition linked to altered hippocampal integrity. Evidence suggests high-intensity interval training (HIIT) enhances hippocampal structure and function, with this form of physical exercise potentially mitigating CUD-related cognitive and mental health impairments.

**Objective:**

To determine the impact of a 12-week HIIT intervention on hippocampal integrity (ie, structure, connectivity, biochemistry) compared with 12 weeks of strength and resistance (SR) training in CUD.

**Design, Setting, and Participants:**

This randomized, single-blind, comparator-controlled clinical trial (Brain Exercise and Addiction Trial [BEAT]) assessed the efficacy of a 12-week exercise intervention for improving hippocampal integrity. Participants were adults with moderate to severe CUD and were not required to cease cannabis consumption. In-person assessments and interventions were conducted at Monash University’s BrainPark facility (Melbourne, Australia). The trial was conducted from 2018 to 2022 and the data analysis from September 2022 to February 2023.

**Intervention:**

HIIT (3 times a week, high lactate condition) for 12 weeks, compared with 12 weeks of SR (3 times a week, low lactate, active control condition) to 12 weeks. Exercise interventions were supervised by exercise physiologists and tailored to target specific (lactate) and personalized physiological mechanisms.

**Main Outcomes and Measures:**

The primary outcome was hippocampal integrity as indicated by a composite of 3 magnetic resonance imaging (MRI) measures: anatomical volume, fractional anisotropy, and N-acetylaspartate. Secondary outcomes included cognitive and mental health measures. Outcomes were assessed at baseline and at the end of the intervention. Adverse events were tracked throughout participation.

**Results:**

Fifty-nine participants with moderate to severe CUD were randomized 1:1 to receive HIIT or SR. The mean (SD) age was 27.0 (6.3) years (range, 20-53 years); 47 participants (80%) were male and 12 (20%) female. Overall, 47 participants (80%) completed the 12-week intervention, attending a mean of 29 of 36 exercise sessions (80%). Hippocampal integrity did not increase after 12 weeks of HIIT (estimated marginal means [SE], −0.14 [0.43] at baseline; 0.10 [0.45] after intervention) or SR (0.38 [0.37] at baseline; −0.16 [0.37] after intervention).

**Conclusions and Relevance:**

This trial found that a 12-week HIIT intervention did not improve hippocampal integrity or associated cognitive or mental health impairments while people continued to consume cannabis. However, results indicated that people with CUD can engage in regular physical exercise programs and highlighted exercise as a potential strategy to reduce cannabis craving.

**Trial Registration:**

ClinicalTrials.gov Identifier: NCT04902092

## Key Points

**Question:**

Does 12 weeks of high-intensity interval training lead to greater improvements in hippocampal health among individuals with cannabis use disorder compared with 12 weeks of strength and resistance training?

**Findings:**

This randomized clinical trial showed that neither high-intensity interval training nor strength and resistance training led to any detectable increases in hippocampal health.

**Meaning:**

High-intensity interval training does not appear to improve hippocampal health or related cognitive functioning and mental health symptoms while cannabis use persists; however, the findings suggest that with appropriate supports, individuals with cannabis use disorder can successfully participate in intensive exercise programs.

## Teaser

This randomized clinical trial examines the effects of a 12-week high-intensity interval training on hippocampal integrity compared with 12 weeks of strength and resistance training among individuals with cannabis use disorder.

## Introduction

Cannabis is the most widely used illicit substance globally.^1^ With increasing legalization and availability, use is rising.^1^ While many individuals use cannabis without major consequences, 10% to 30% of regular users develop cannabis use disorder (CUD), characterized by persistent use despite negative consequences, including mental ill health (eg, low mood, anxiety, apathy) and cognitive impairments (eg, impaired learning and memory). These adverse effects are linked to cannabis-induced structural and functional alterations in the hippocampus.^2^

The hippocampus is particularly susceptible to prolonged cannabis exposure.^3^ Neuroimaging studies have linked CUD to reductions in hippocampal integrity (eg, structure, connectivity, biochemistry).^3,4^ As a critical region for learning, memory, and emotional processing,^5^ hippocampal integrity is functionally related to many of the brain-based symptoms of CUD, including poor verbal learning and memory, mood disturbances, anxiety, and apathy.^6^ Despite these consequences, no interventions currently target hippocampal integrity in CUD.

Cardiovascular exercise, particularly high-intensity interval training (HIIT), is a potent modulator of neuroplasticity and hippocampal integrity.^7^ HIIT induces significant blood lactate production, which crosses the blood-brain barrier to stimulate brain-derived neurotrophic factor (BDNF),^8^ a key regulator of neuronal growth and connectivity. Evidence suggests HIIT can enhance hippocampal volume, connectivity, and function in healthy and clinical populations.^9^ Cardiovascular exercise can also reduce craving, a predictor of relapse,^10^ in substance use disorders and improve cognition impairments that complicate treatment adherence. However, its effects on individuals with CUD remain unexplored.

Few studies have precisely tailored exercise interventions to target specific physiological mechanisms (ie, low vs high lactate exposure) or comprehensively assessed hippocampal integrity using multimodal imaging. Adherence to exercise interventions in substance-using populations is also challenging, necessitating a behaviorally informed approach. Supervised, group-based interventions led by exercise professionals may optimize engagement and offer valuable insights into the impact of exercise on CUD.

This trial examined the effects of a 12-week HIIT intervention (high lactate exposure) vs strength and resistance (SR) training (low lactate exposure) on hippocampal integrity in people with moderate to severe CUD. The hypothesis was that 12 weeks of HIIT would produce greater improvements in hippocampal integrity than SR training, offering a novel, behaviorally informed approach to addressing neurobiological deficits in CUD.

## Methods

### Trial Design

**Figure 1.**
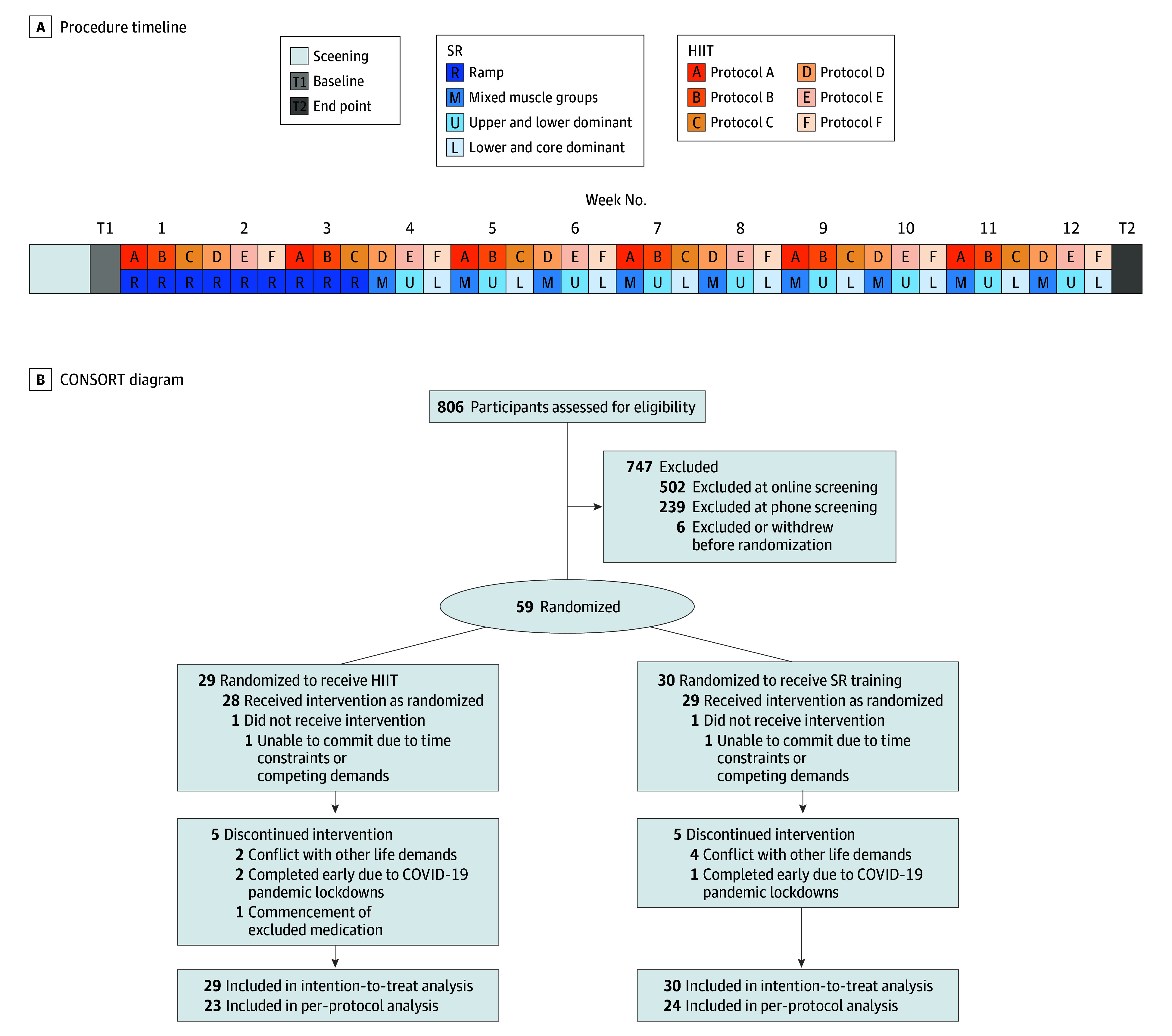
Procedure Timeline and Study Flow Diagram A, After the screening procedures and baseline assessment, participants attended 36 high-intensity interval training (HIIT) or strength and resistance (SR) training sessions over 12 weeks, followed by the end point assessments. B, Participant numbers and disposition through the course of the trial.

This randomized clinical trial (NCT04902092) using a 1:1 ratio (stratified by dependence severity, age, and gender) and a parallel design was approved by the Monash University Human Research Ethics Committee (Figure 1). (See Supplement 1 for the full trial protocol.) Participants were blind to the hypothesized superiority of HIIT but not to exercise condition. All participants provided written informed consent. This article adheres to the Consolidated Standards of Reporting Trials (CONSORT) and Template for Intervention Description and Replication (TIDieR) reporting guidelines for improved reporting of randomized trials of nonpharmacological treatments. The trial was conducted from 2018 to 2022 and the data analysis from September 2022 to February 2023.

### Participants

Inclusion criteria included moderate to severe CUD, age 20 to 55 years, and a significant history of cannabis use (more than 3 days per week for 4 of the past 6 years). Exclusion criteria included significant polysubstance use, comorbid mental illness, or contraindication to magnetic resonance imaging (MRI), cardiopulmonary exercise testing (CPET), or regular physical exercise.

### Intervention

Both exercise conditions involved three 45-minute sessions per week for 12 weeks. Participants wore a heart rate monitor so accredited exercise physiologists could adjust exercise intensity in real time based on individual needs. The HIIT protocol was designed to increase lactate exposure, while SR sessions aimed to minimize it (eFigures 1-6, eTables 3-5, and the eMethods in the Supplement 2 and eTables 1 and 2 in Supplement 3). Accredited exercise physiologists provided motivational support tailored to participants’ barriers and facilitators to maximize adherence.

### Outcome Measures

All outcomes were collected at baseline and within 1 week of concluding the intervention. The primary outcome was left hippocampal integrity, a composite of hippocampal volume, fractional anisotropy, and N-acetylaspartate obtained on a 3-Tesla Siemens Skyra MRI scanner (eFigure 7 in Supplement 2).

### Secondary Outcomes

Secondary outcome measures assessed cannabis use (craving, consumption, dependence), cognition (visual learning and memory, paired associate learning), mental health (apathy, anxiety, depression, resilience, well-being, quality of life, sleep), physical activity, and cardiorespiratory fitness using validated measures.

### Statistical Analysis

Analyses were performed using SPSS version 26.0 (IBM). Intent-to-treat and per-protocol analyses were conducted. Main effects and group × time interactions were assessed using generalized estimating equations. Covariates included age, gender, age of regular cannabis use, and cannabis consumption (grams). The α was 2-sided and set at .01.

## Results

**Figure 2.**
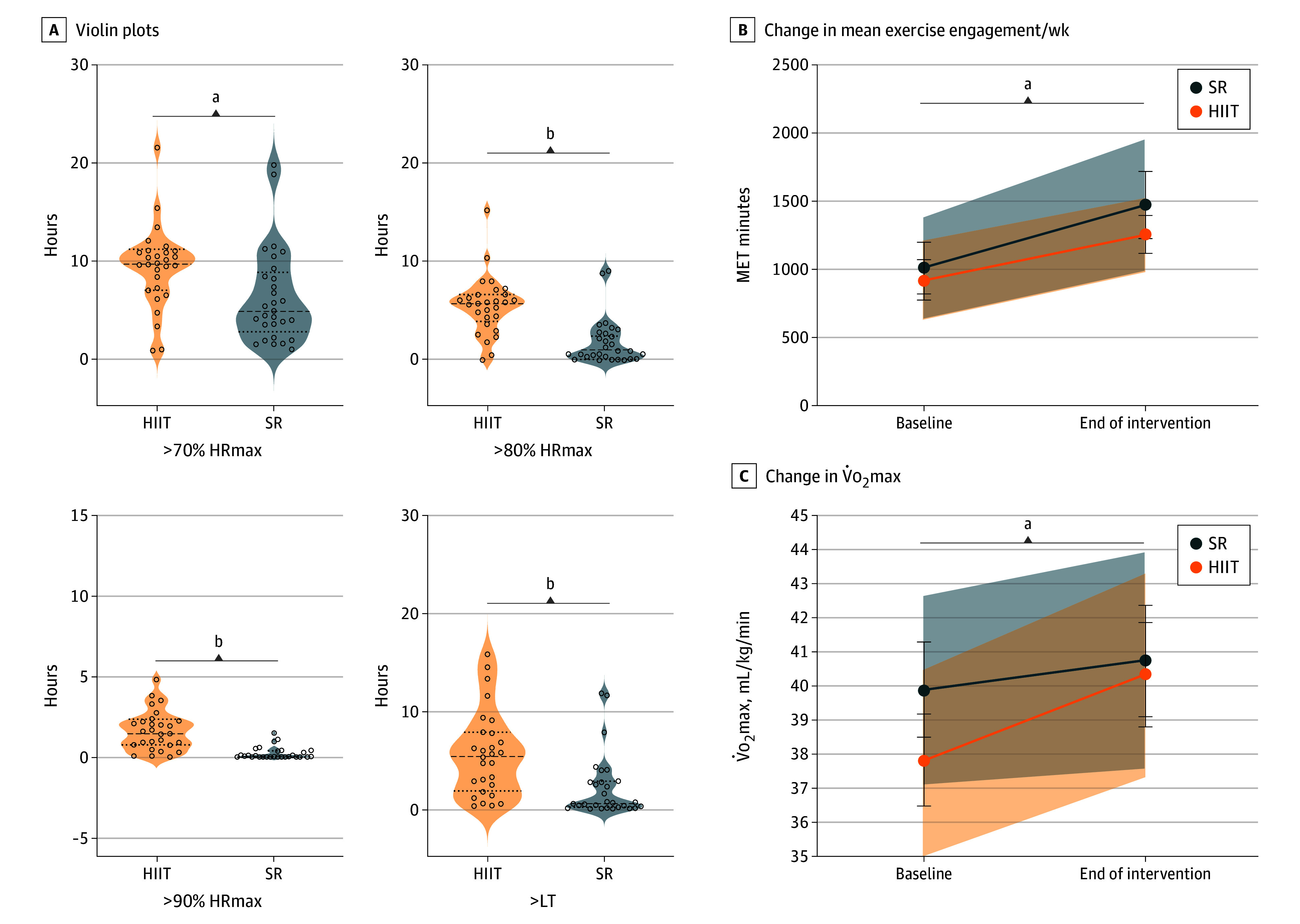
Exercising Time, Engagement, and Exertion in the High-Intensity Interval Training (HIIT) and Strength and Resistance (SR) Training Groups A, Time spent exercising above 70%, 80%, and 90% of maximum heart rate (HRmax) and lactate threshold (LT). B, Change in mean exercise engagement per week (metabolic equivalent [MET] minutes) for participants in the HIIT and SR groups from baseline to end point (intent to treat). C, Change in maximum oxygen consumption (V̇o_2_max) for participants in the HIIT and SR groups from baseline to end point (per protocol). Error bars represent standard error; dashed lines and shading indicate 95% CIs. ^a^*P* < .01. ^b^*P* < .001.

Fifty-nine participants completed the baseline assessment. The mean (SD) age was 27.0 (6.3) years (range, 20-53 years); 47 participants (80%) were male and 12 (20%) female (eTable 4 in Supplement 2). Forty-seven completed the intervention (79.7%), attending 80% of exercise sessions. The HIIT group spent significantly more time exercising above 70% of maximum heart rate (HRmax) (*U* = 231.5, *z* = −2.79; *P* = .005), 80% HRmax (*U* = 121.5, *z* = −4.54; *P* < .001), 90% HRmax (*U* = 88.5, *z* = −5.10; *P* < .001), and the lactate threshold (*U* = 172.0, *z* = −3.74; *P* < .001) (Figure 2). Mean exercise engagement increased significantly from baseline to end point, regardless of condition (Wald χ^2^ = 5.199; *P* = .02) (Figure 2). No change in maximum oxygen consumption (V̇o_2_max) was seen in the intent-to-treat analysis; however, the per-protocol analysis revealed a greater increase in V̇o_2_max for the HIIT group vs the SR group (Figure 2).

### Primary Outcome

**Figure 3.**
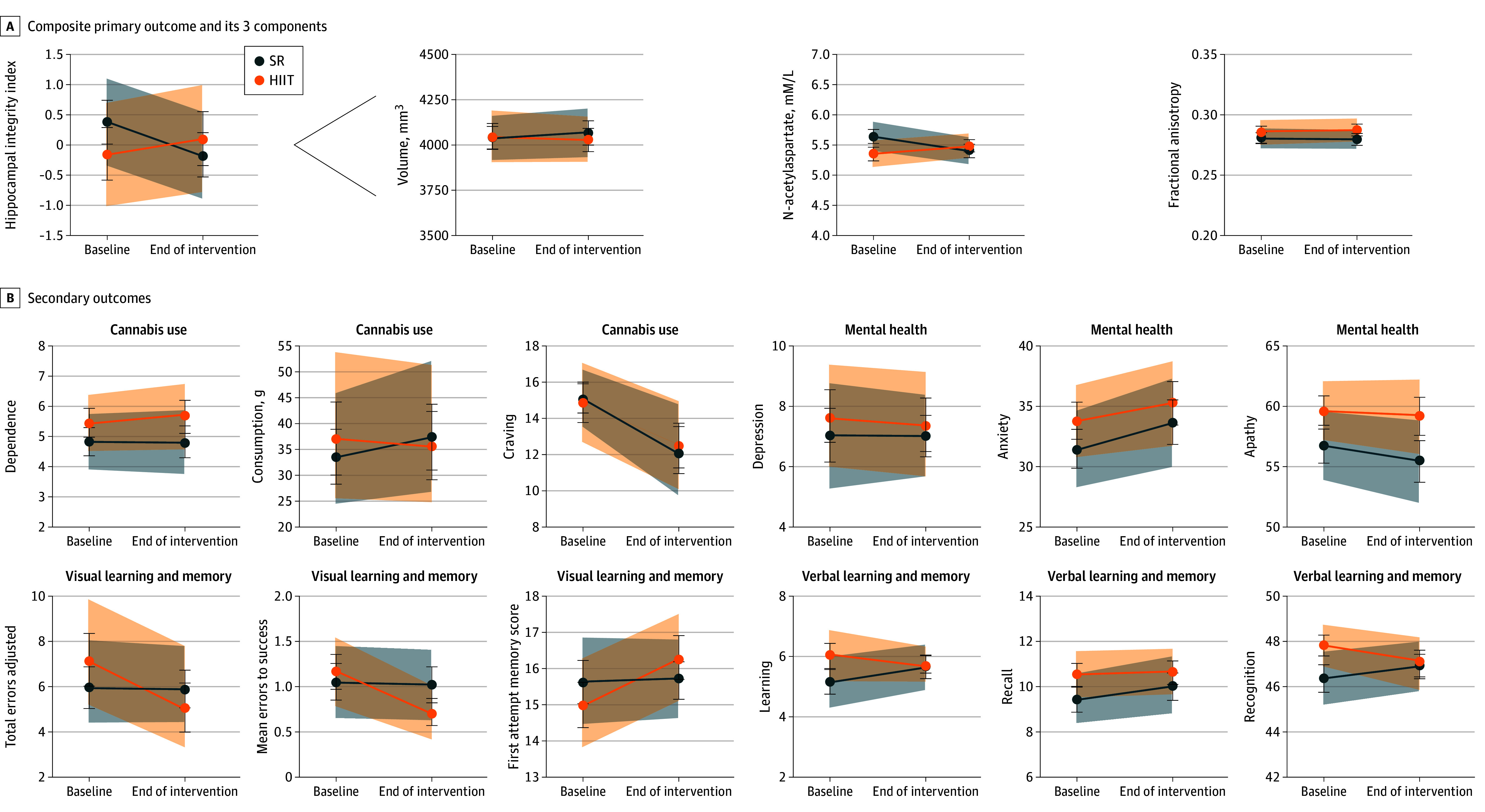
Estimated Marginal Means (EMM) for Participants From Generalized Estimating Equation (GEE) Regression on Primary and Secondary Outcome Measures A, EMM for participants in the high-intensity interval training (HIIT) and strength and resistance (SR) training groups from corresponding intent-to-treat (ITT) GEE regression on hippocampal integrity, left hippocampal volume, N-acetylaspartate, and fractional anisotropy. B, EMM for participants in the HIIT and SR groups from corresponding ITT GEE regressions on substance use, mental health, and verbal and visual memory function. Error bars represent standard error; dashed lines and shading indicate 95% CIs. Cannabis dependence was assessed by Severity of Dependence Scale; consumption, timeline follow-back procedure; craving, Penn Cannabis Craving Scale; depression, Quick Inventory of Depressive Symptomatology; anxiety, State-Trait Anxiety Inventory–state anxiety scale; apathy, Apathy Evaluation Scale; visual learning and memory, paired associate learning; and verbal learning and memory, Rey Auditory Verbal Learning Test.

Hippocampal integrity did not increase after 12 weeks of HIIT (estimated marginal means [EMM] [SE], −0.14 [0.43] at baseline; EMM [SE], 0.10 [0.45] after intervention) or SR training (EMM [SE], 0.38 [0.37] at baseline; EMM [SE], −0.16 [0.37] after intervention), when hippocampal integrity was examined as a composite or by individual components (Figure 3A and eTables 6 and 7 in Supplement 2).

### Secondary Outcomes

Per-protocol analysis revealed a significant reduction in craving for both conditions (Wald χ^2^ = 6.04; *P* = .01). No other secondary outcome measures changed (Figure 3B and eTables 8-10 and eFigures 8-11 in Supplement 2).

## Discussion

This randomized clinical trial investigated whether HIIT improves hippocampal integrity in people with CUD. Contrary to expectations, HIIT did not enhance hippocampal integrity, cognitive function, or mental health over time or compared with SR training, despite high engagement and a behaviorally and physiologically informed intervention design. The lack of effect over time may stem from cannabis-related neurobiological alterations, including tetrahydrocannabinol (THC) accumulation within neurons, altered cannabinoid receptor signaling, and vascular and dendritic impairments. Continued cannabis use during the trial may have interfered with exercise-induced neuroplasticity. Animal studies suggest THC reduces BDNF expression, a key driver of hippocampal neuroplasticity.^11^ As such, abstinence may be necessary to observe meaningful neurobiological changes.

It is also possible that both HIIT and SR training conferred benefits that may have reduced detectable differences between groups, as supported by an association between training frequency and hippocampal volume and fractional anisotropy that is independent of training condition. SR training is known to enhance neurotrophic factors and brain health, potentially mitigating the expected superiority of HIIT. Additionally, maintaining hippocampal integrity amid ongoing cannabis use and pandemic-related stressors may reflect a protective effect of exercise. Future research should investigate whether exercise prevents the decline of hippocampal integrity in people who continue to consume cannabis using a waitlist or inactive control condition.

Both interventions reduced cannabis craving, a known predictor of relapse, over time, supporting previous research on the role of exercise in addiction treatment.^12,13^ Potential mechanisms include shared activation of reward pathways and improved mood and stress regulation. Engagement was exceptionally high, with 80% adherence, surpassing previous addiction-related exercise interventions.^13^ The supervised, group-based structure likely contributed to this success. Future studies should examine whether exercise enhances hippocampal integrity when combined with abstinence strategies.

### Limitations

Trial limitations include an overrepresentation of male participants, small sample size, and COVID-19 disruptions. In addition, conclusions are specific to the 12-week trial duration. Nonetheless, this trial highlights exercise as a promising strategy to reduce cannabis craving, with further research needed to explore gender differences and determine its effects on hippocampal integrity after abstinence.

## Conclusions

This trial found that a 12-week HIIT intervention did not improve hippocampal integrity or associated cognitive or mental health impairments while people continued to consume cannabis. However, results indicated that people with CUD can engage in regular physical exercise programs and highlighted exercise as a potential strategy to reduce cannabis craving.

## Notes

Supplement 1. Trial protocol

Supplement 2.
eMethods
**eFigure 1.** HIIT Protocol A: Mixed Functional Exercises **eFigure 2.** HIIT Protocol B: Mixed Functional Exercises **eFigure 3.** HIIT Protocol C: Mixed Functional Exercises **eFigure 4.** HIIT Protocol D: Mixed Functional Exercises **eFigure 5.** HIIT Protocol E: Mixed Functional Exercises **eFigure 6.** HIIT Protocol F: Mixed Functional Exercises **eTable 3.** Strength and Resistance Protocol: Upper and lower muscle groups template **eFigure 7.** Hippocampal integrity
eResults
**eTable 4.** Demographic and Baseline Characteristics **eTable 5.** Exercise engagement between participant who did, or did not have at least one exercise session via video-call. **eTable 6.** Primary Outcome GEE Regression Results from ITT analysis. **eTable 7.** Primary Outcome GEE Regression Results from per protocol analysis **eTable 8.** Secondary Outcomes GEE Regression Results from ITT analysis **eTable 9.** Secondary Outcomes GEE Regression Results from PP analysis **eTable 10.** Linear regression model for primary outcome measures **eFigure 8.** Estimated marginal means for participants in the HIIT and S&R condition from corresponding ITT GEE regressions for resilience, wellbeing, quality of life, and sleep **eFigure 9.** Means successful memory retrieval on the FigMem task for participants in the HIIT and S&R condition **eFigure 10.** Brain activation during memory successful retrieving phase of the FigMem Task **eFigure 11.** Brain activation during memory encoding phase (contrasting with resting) of the FigMem Task
eReferences

Supplement 3. **eTable 1.** HIIT exercise library **eTable 2.** Strength and Resistance exercise library

Supplement 4. Data sharing statement

## References

[1] Connor JP, Stjepanović D, Le Foll B, Hoch E, Budney AJ, Hall WD. Cannabis use and cannabis use disorder. Nat Rev Dis Primers. 2021;7(1):16. doi:10.1038/s41572-021-00247-4 33627670 PMC8655458

[2] Chye Y, Lorenzetti V, Suo C, . Alteration to hippocampal volume and shape confined to cannabis dependence: a multi-site study. Addict Biol. 2019;24(4):822-834. doi:10.1111/adb.12652 30022573

[3] Yücel M, Lorenzetti V, Suo C, . Hippocampal harms, protection and recovery following regular cannabis use. Transl Psychiatry. 2016;6(1):e710-e710. doi:10.1038/tp.2015.201 26756903 PMC5068875

[4] Yücel M, Solowij N, Respondek C, . Regional brain abnormalities associated with long-term heavy cannabis use. Arch Gen Psychiatry. 2008;65(6):694-701. doi:10.1001/archpsyc.65.6.694 18519827

[5] Jager G, Van Hell HH, De Win MML, . Effects of frequent cannabis use on hippocampal activity during an associative memory task. Eur Neuropsychopharmacol. 2007;17(4):289-297. doi:10.1016/j.euroneuro.2006.10.003 17137758

[6] Leuner B, Gould E. Structural plasticity and hippocampal function. Annu Rev Psychol. 2010;61(1):111-140, C1-C3. doi:10.1146/annurev.psych.093008.100359 19575621 PMC3012424

[7] Kandola A, Hendrikse J, Lucassen PJ, Yücel M. Aerobic exercise as a tool to improve hippocampal plasticity and function in humans: practical implications for mental health treatment. Front Hum Neurosci. 2016;10:373. doi:10.3389/fnhum.2016.00373 27524962 PMC4965462

[8] Müller P, Duderstadt Y, Lessmann V, Müller NG. Lactate and BDNF: key mediators of exercise induced neuroplasticity? J Clin Med. 2020;9(4):1136. doi:10.3390/jcm9041136 32326586 PMC7230639

[9] Hendrikse J, Chye Y, Thompson S, . Regular aerobic exercise is positively associated with hippocampal structure and function in young and middle-aged adults. Hippocampus. 2022;32(3):137-152. doi:10.1002/hipo.23397 34961996

[10] Vafaie N, Kober H. Association of drug cues and craving with drug use and relapse: a systematic review and meta-analysis. JAMA Psychiatry. 2022;79(7):641-650. doi:10.1001/jamapsychiatry.2022.1240 35648415 PMC9161117

[11] Winstone J, Shafique H, Clemmer ME, Mackie K, Wager-Miller J. Effects of tetrahydrocannabinol and cannabidiol on brain-derived neurotrophic factor and tropomyosin receptor kinase B expression in the adolescent hippocampus. Cannabis Cannabinoid Res. 2023;8(4):612-622. doi:10.1089/can.2021.0025 35639364 PMC10442678

[12] Pechtl SML, Abrantes AM, Sjöqvist H, Andreasson S, Herring MP, Hallgren M. Do changes in mood and anxiety mediate exercise-induced reductions in alcohol cravings? an exploratory study. Psychol Addict Behav. 2024;38(8):827-835. doi:10.1037/adb0000987 38252110

[13] Türkmen C, Martland R, Grilli M, Stubbs B, Roessler KK, Hallgren M. Can high-intensity interval training improve health outcomes among people with substance use disorders? a systematic review and preliminary meta-analysis. Ment Health Phys Act. 2024;27:100622. doi:10.1016/j.mhpa.2024.100622

