# Supplementary material for: High-Intensity Exercise and Hippocampal Integrity in Adults With Cannabis Use Disorder: A Randomized Clinical Trial
Source: JAMA Psychiatry. 2025 Sep 10;82(12):1240–5. doi: 10.1001/jamapsychiatry.2025.2319 (PMC12423954; doi:10.1001/jamapsychiatry.2025.2319)
Supplement: Supplement 1. — Trial protocol [file jamapsychiatry-e252319-s001.pdf]

Brain Exercise and Addiction Trial: Efficacy of a 12-week aerobic exercise regime for restoring 'brain health' in cannabis users

Synopsis

Over the past decade, the CI team have demonstrated that heavy cannabis use is associated with substantive learning and memory impairments and elevated risk of psychopathology. We have repeatedly demonstrated that the hippocampus, centrally implicated in these processes, is particularly vulnerable to the deleterious effects of prolonged exposure to cannabis. We have now shown that this deterioration of hippocampal structure, function, and biochemistry can be reversed, but this requires two or more years of abstinence from cannabis. However, most heavy cannabis users find it extremely difficult to maintain abstinence over extended periods and current treatments for cannabis use disorders are inadequate. There is a pressing clinical need for an intervention that rapidly accelerates hippocampal recovery, ameliorates the associated cognitive impairments and mental health symptoms, and leads to improved treatment outcomes. Regular aerobic exercise, specifically High Intensity Interval Training (HIIT), has emerged as a simple and safe yet powerful candidate to effectively reduce cannabis use/craving and promote hippocampal health, and improve cognition and well-being. We have recently conducted a study of 12-weeks HIIT in healthy adults and demonstrated positive changes in hippocampal biochemistry (15% increase in metabolites indexing neuronal integrity), and structure (3% increase in volume) induced by exercise, as well as improved learning and memory capacity. These brain and cognitive improvements *were not* observed with progressive strength and resistance training, suggesting that the effects are dependent on a specific (i.e., HIIT) exercise regime. In the current proposal, we will recruit individuals with a long-term and heavy history of cannabis use into either a 12-weeks supervised HIIT intervention or an active control condition, allowing us to determine whether HIIT can expedite hippocampal recovery, reduce cannabis use, ameliorate psychopathology, and improve cognitive and general well-being.

Background

Australia has one of the highest rates of cannabis use in the world. Within the 2010 National Drug Strategy Household Survey, ~9 million Australians (35%) reported using cannabis at some time in their lives. While most use the drug infrequently, ~500,000 report using at least weekly [1]. Despite a low mortality rate, cannabis use has broad ranging and significant adverse health effects including heightened risk of developing psychotic symptoms [2], and impairments in learning, memory, and socio-educational achievements [3-6]. Unfortunately, there are currently no evidence-based pharmacotherapy treatments for cannabis use disorders or cannabis withdrawal [7], and behavioral therapies are often either not accessible or over-stretched [8]. **CI-Yücel**, **Solowij**, and **Lubman's** work has consistently revealed that long-term use is linked with structural and functional degradation of the hippocampus, cognitive impairment, and poor clinical outcomes [3, 9-14].

Hippocampal harms are robustly linked to regular and heavy cannabis use

In a series of world-first studies, we have demonstrated the links between long-term cannabis use and hippocampal harms, including: (i) a dose-dependent reduction in hippocampal *volume* [13]; a large reduction in *connectivity* and axonal fibre integrity in the hippocampal region [12]; and (iii) lower *biochemical* concentrations of hippocampal n-acetylaspartate (NAA), reflecting reduced neuronal integrity [14]. These findings have established the CIs as world leaders in cannabis research. Many of them have been independently replicated [15-17]. Collectively, our results reveal the negative long-term and persistent effects of regular cannabis use on neurobiological and psychosocial outcomes. *It remains unconfirmed whether these hippocampal alterations can be reversed, but we have compelling pilot data demonstrating that they may be.*

Fig. 1: *Plots show hippocampal: (a) volume; and (b) n-acetylaspartate levels in long-term cannabis users (N=30; green), former users (N=12; blue/***arrow***), and controls (N=37; red). Horizontal lines represent group means and 95% confidence intervals. *p<.05; **p<.005. Data corrected for intracranial volume, age, IQ, anxiety and depressive symptoms, alcohol and tobacco use [14].*

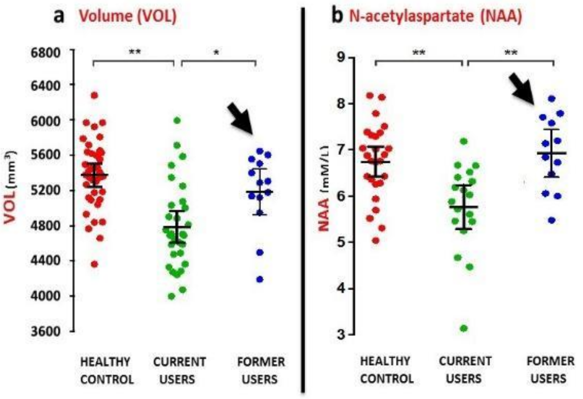

PILOT STUDY 1: Hippocampal integrity may be recovered through prolonged abstinence

Our team recently generated promising findings for recovery of hippocampal volume and biochemistry in former users (**Fig. 1**). Specifically, former-users matched on exposure to current users, who were abstinent for ~2 years, showed hippocampal volume and NAA levels that were significantly ($p<0.05$) higher than current users and comparable to non-using healthy controls.

These world-first data suggest that recovery can occur over an extended period of reduced exposure to cannabis. However, only a very small proportion of heavy users manage to cease use for prolonged periods of up to 2 years [18]. This inability to sustain abstinence may be partially underpinned by the fact that the deleterious effects of cannabis on the hippocampus have been linked to increased depressive and psychotic symptoms, as well as impaired learning and memory [19]. As such, the triad of hippocampal harms, cognitive impairments, and mental health symptoms may serve to maintain cannabis use, and lead to increased rates of relapse and/or decreased abstinence. While our findings strongly suggest that hippocampal harms are modifiable, alternative approaches to accelerate recovery or minimise the harms of ongoing use are vital.

Physical exercise is a simple intervention with the potential to enhance hippocampal recovery

Normative studies, including that by Erickson *et al.* (2011), have shown that hippocampal volume in the elderly can be ‘preserved’ through regular brisk walking [20] and that hippocampal volume is larger in physically “fit” individuals, protecting them against future age-related volume loss by stimulating neurotrophins, plasticity, and possibly, neurogenesis [21]. Hippocampal volume can increase in young adults following as little as 6-weeks exercise [22], however this 6 weeks training effect is transient and not associated with enhanced cognition, suggesting longer interventions are necessary. Substantial evidence supports a 3-month timeframe for more sustained effects of exercise interventions. For instance, 3-months of exercise has been shown by Pajonk and colleagues to be sufficient to produce a 16% and 12% increase in hippocampal volume of healthy young adults and schizophrenia patients respectively, together with improved cognition and mental health [23].

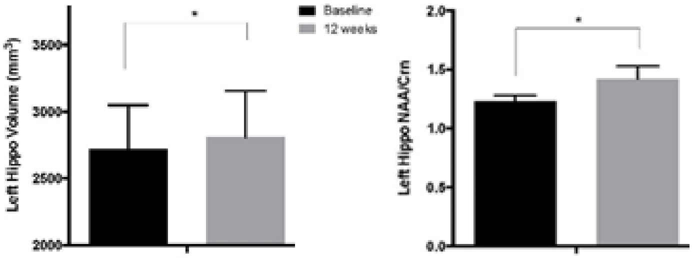

Fig. 2: *Hippocampal changes after 12-weeks of HIIT* in our sample of healthy adults (N=10; 39.7±10 years) showing increased volume (3%; *p*=.034) and NAA levels (15%; *p*=.004).

In addition, High Intensity Interval Training (HIIT) has been shown to be especially effective in driving structural and functional changes in the brain, particularly in the hippocampus [22, 24]. HIIT involves a short burst of high-intensity exercise followed by a brief low-intensity activity, repeatedly, until the individual has exceeded their ability to maintain a steady state. The powerful effect HIIT has on the hippocampus is thought to be due to the high lactate state induced by this form of training. Lactate is a by-produce released during glycolysis, the process through which glucose is broken down to produce energy during anaerobic activity such as HIIT. During bouts of HIIT, high amounts of lactate cross the blood brain barrier, where it acts as a signalling molecule, triggering the production of brain-derived neurotrophic factor (BDNF) (Mueller et al., 2020). BDNF is responsible for the regulation of multiple neurotrophic processes such as promoting overall neuronal health, neurotransmission and neurogenesis (Cassilhas et al., 2015; El-Sayes et al., 2019; Erickson et al., 2010; Park & Poo, 2013).

<u>PILOT STUDY 2</u>: HIIT exercise improves hippocampal integrity

Extending upon the findings of the Pajonk study, we now have two preliminary datasets in healthy volunteers showing: (i) the positive impact of a 3-month HIIT programme on brain volume and biochemistry (see **Fig. 2**), as well as cognition (i.e., learning and memory); and (ii) the lack of any changes after *progressive strength and resistance training* (N=39; data not shown).

Together, these findings strongly support our prediction of a 3-month HIIT intervention to induce positive hippocampal changes, and our design of using low-moderate intensity training (e.g strength and resistance) as an active control. More broadly, three small-scale

studies by other investigators have documented that regular exercise has beneficial effects on drug intake and mental health in current drug users [29-31]. The study of Buchowski *et al.* (2011) is particularly striking, showing that a short course of HIIT reduces cannabis craving and use in dependent, non-treatment seeking adults [30]. Stronger effects would be expected in those motivated to change their behaviour, requiring confirmation using a rigorous experimental design in a larger sample.

Research Plan

We have reliably demonstrated that the hippocampus is deleteriously affected by regular and heavy cannabis use [3, 9-14]. We now have pilot evidence showing that reduced exposure to cannabis for prolonged periods promotes recovery of the hippocampus. We also have data highlighting the potential for accelerated hippocampal recovery following a 3-month HIIT program, which we hypothesise will also be observable in our population of help-seeking cannabis users and associated with beneficial effects on cognition, mental health and general well-being. Small-scale reports also suggest that exercise can reduce cannabis craving and use. Based on these findings, we predict:

<u>Primary hypothesis (outcome at 12-weeks relative to baseline):</u>

1. Cannabis using individuals in the HIIT program (EXE+) will have greater improvement in *hippocampal integrity* (a composite score of volume, fibre density, and NAA) than those in the low-moderate intensity strength training exercise program (EXE–; active control).

<u>Secondary hypotheses (outcomes at 3- and 9-months relative to baseline)</u>:

2a. Cannabis users in the EXE+ will have reduced cannabis use (freq. of use, % of abstinence days) and psychopathology (depressive and psychotic symptoms), as well as improved cognition (learning, memory) and general well-being (social and occupational functioning).

2b. Any improvements in Hypothesis 2a will be mediated by changes in hippocampal integrity.

Method

Participants

One hundred and fifty cannabis users (aged 20 – 55 years) from the community and referred from Turning Point, a national service provider that engages with thousands of cannabis users each year, whom are seeking to reduce their cannabis use but are not currently enrolled in treatment, will be recruited into the study.

Inclusion criteria:

1. Aged 20-55 years
2. Voluntary and able to provide informed consent
3. Fluent in English
4. Current moderate – severe cannabis use disorder

5. Major history of cannabis use (i.e. ≥3 days per week on average for ≥4 of the past 6 years)

Exclusion criteria:

1. Current engagement in CUD treatment,
2. Presence of a neurological disorder or serious head injury;
3. Lifetime history of bipolar disorder, obsessive-compulsive disorder, post-traumatic stress disorder, psychosis or autism spectrum disorder;
4. Current, unstable use of psychotropic medication;
5. Contraindications for MRI (i.e., metal implants and claustrophobia);
6. Current unstable, or chronic medical illness (i.e., cardiovascular disease, chronic pain, musculoskeletal injury), that would preclude safe engagement in CPET and/or regular physical exercise;
7. Engaging in shift work in the previous six months;
8. Engaging in five or more HIIT sessions within the previous six months;
9. Moderate and/or severe substance use disorder other than CUD;
10. Pregnancy or lactation.
11. Other psychoactive medications or psychosocial treatments will be considered on a case-by-case basis. Where a current psychoactive medication is deemed acceptable, both dose and type must have been stable for a minimum of four weeks prior to baseline assessment, and remain stable throughout the 12-week exercise phase of the study.

Please note, only individuals who are voluntary, able to provide full informed consent and able to adhere to the study procedures will be recruited.

These criteria have been designed to exclude individuals for whom the protocol may pose a risk to physical safety (e.g. history of cardiovascular or musculoskeletal injury, contraindication to MRI) and control for variables which would confound the study results (e.g. neurological abnormalities on brain imaging, pre-existing cognitive impairment on cognitive testing).

Procedure

The study will employ a single-blind, active-controlled, parallel group randomised design. Participants will be randomised to engage in a 12-weeks program of one of the following two physical training conditions:

a. EXE+ condition: 12-weeks of HIIT
b. EXE- condition: 12-weeks of strength training

Following recruitment and written informed consent, participants will be randomly allocated to the study groups in a 1:1 ratio using permutated blocks of random size generated by a statistician not involved in the study. Group allocation will be stratified to balance cannabis dependence severity (according to the severity of dependence scale (SDS)) and sex. We will

also employ post-stratification assessments of key variables at the analysis stage (including age, baseline fitness, and nicotine use). Treatment allocation concealment will be achieved by using a password protected computer program, implemented by an independent member of the Monash University administration team who will inform the research team about the randomisation outcome. Following randomisation, participants will be informed about the assessment protocols, and the EXE program.

Outcome measures will assess psychological (questionnaires), biological (MRI, MRS, urine assays, hair and saliva assays), cognitive (hippocampal function), cannabis use (questionnaires) levels of fitness (VO2 test), and physical activity (heart-rate physical activity via Garmin device). Primary assessments will be made at baseline, end of 12-week and 6-month post-intervention follow up review. Brief assessment of psychological wellbeing and cannabis use will be made weekly via the Qualtrics smartphone platform. The intervention and assessment schedule is depicted below in Table 1.

Table 1. Participant Timeline of Testing Procedures

Pre-training	12-week training	Post-training	6-month follow up
MRI brain scan	3x weekly exercise	MRI brain scan	Questionnaires
Questionnaires	1x weekly phone call and questionnaires	Questionnaires	Cognitive activities
Cognitive activities		Cognitive activities	
Hair, urine and saliva samples		Hair and urine samples	
CPET		CPET	

Intervention

Core exercise intervention design features aim to achieve two key physiological and behavioral outcomes:

I. Group differentiation in expected blood lactate accumulation, using heart rate as a proxy indicator (i.e.: HIIT condition = higher blood lactate accumulation; Strength condition = lower blood lactate accumulation).
II. Mechanisms to increase engagement and retention (i.e.: exercise variety and personalization elements).

Both exercise interventions will take 12-weeks to complete, with a weekly frequency of 3 exercise sessions per week, session duration will be 45 minutes. All sessions will be supervised by accredited exercise physiologists and conducted at BrainPark. To segregate interventions, HIIT and Strength sessions will occur at separate times of day. Exercise sessions will be conducted in groups of up to 6 participants. Participants will be given a Polar

heart rate monitoring device to wear during each session. A 5 minute warm up period of moderate subjective intensity will occur at the beginning of each session, across both conditions, followed by:

EXE+ Protocol: HIIT

The six HIIT sequences will contain an accumulative 12 minutes of high-intensity work time (i.e.: target ≥ 80% of a peak heart rate determined from baseline CPET). Depending on the session variation undertaken, high-intensity intervals range from 1 minute to 4 minutes with rest periods at, or below, the same work interval duration - equating to a work to rest ratio of ≥1:1. There is flexibly on how rigid this target is applied and depends on the psycho-physical characteristics of the participant (e.g.: confidence, exercise tolerance and motor skill abilities) and also stage of intervention (i.e.: more flexibility is given in the first 3 weeks of the intervention to develop exercise tolerance and to apply principles of progressive overload).

EXE- Protocol: Strength training

Participants will undertake strength training predominantly targeting large muscle groups, the target intensity range will be low-moderate (i.e.: target ≥ 50% - < 80% of peak CPET heart rate). Strength training will involve 12-14 sets of 5-12 repetitions, across 6 individual strengthening movements (i.e.: 2-3 sets per movement). Throughout the 12-weeks participants will rotate between 3 session variations, each with variations in muscle-group focus:

1. Upper and lower body muscle groups;
2. Core and lower and
3. Mixed session (upper, core and lower).

Like the HIIT condition, there is flexibility in the program design to cater for disparities in psycho-physical characteristics, for instance over the 12-weeks principles of progressive overload will be applied on an individual basis at the discretion of the exercise physiologists (eg: rate of progression). Sessions finish with a 5 minute cool-down period of light intensity involving stretching and debriefing (see Table 2 for further design components).

Table 2. Exercise Intervention Components

Component	HIIT	Strength
Weeks	21	
Frequency (sessions per week)	3/7	
Session duration (minutes per session)	45 minutes	
Session time structure	5mins - Warm up period	5mins - Warm up period

	20-25mins - HIIT period<br>15-20mins - Cool down period	35mins - Strength period<br>5mins - Cool down period
Accumulative HR targets (%HRpeak per session)	12 mins ≥ 80%<br>33 mins ≥ 50% - < 80%	45 mins ≥ 50% - < 80%
Interval Work-Rest Ratio	≥ 1:1	N/A
Strength Session Volumes (per session)	N/A	6 movements<br>12-14 sets<br>5-12 repetition range
Session Variations	x 6 HIIT sequences (work:rest ratio minutes)<br>A. [2 x 3:3] + [2 x 2:2] + [1:1]<br>B. [5 x 2:2] + [2 x 1:1]<br>C. [2 x 3:3] + [3 x 2:2]<br>D. 3 x 4:3<br>E. 4 x 3:2<br>F. 6 x 2:2	x 3 Strength programs (muscle-group focus)<br>A. Upper and lower<br>B. Core and lower<br>C. Mixed (upper, lower & core)
Equipment	Cardio-machines; boxing; body-weight; free-weights	Body-weight; free-weights

Blinding (low-level partial deception)

A low-level partial deception will be employed when explaining the purpose of the study. With behavioral interventions, even more so than pharmacological interventions, expectation of positive outcomes is a potent predictor of an individual reporting positive outcomes. HIIT has become very popular and attracted a lot of media interest around positive brain and mental health effects. Thus, advertising the focus on HIIT may lead to disappointment in those individuals randomised to the comparator condition, and disparity between the groups regarding expectation of positive outcomes. These kind of engagement and expectation confounds are barriers to a balanced investigation into the therapeutic potential of HIIT and can contribute to an overestimation of its potency.

As such, rather than describing the focus of the study on HIIT, all participants will be informed that the study is comparing the impact of two different types of physical exercise:

1. Interval training, focused on cardiorespiratory capacity (i.e. HIIT),
2. Strength training, focused on musculoskeletal capacity (i.e. active exercise control).

These are both accurate descriptions of the two training conditions. Both exercise conditions provide an opportunity to improve physical health and fitness and it is not anticipated that this aspect of the study will cause distress. A comprehensive description of the time

commitment and the potential risks and benefits is provided in the explanatory statement, the use of this partial deception does not impact upon these.

Outcome Measures

Demographics

Demographic variables and potential covariates will be recorded at baseline and include:

- Age
- Gender
- Years of education
- Age first tried cannabis
- Age of onset of regular cannabis use
- History of physical and mental ill health
- Family history of cannabis use

Clinical

The Mini International Neuropsychiatric Interview 7.0.2 is a semi-structured diagnostic interview for DSM-V and will be administered by a trained member of the research team. It will be used to screen for current and past mental illness to assess comorbidity. During the telephone screen phase, the MINI screen will be administered to identify those who meet inclusion/exclusion criteria.

Questionnaires/Interviews

Screening

- Physical Activity Readiness Questionnaire (9-item)

Cannabis Use

- Timeline Follow Back Procedure (3-months)
- Severity of Dependence Scale (5-item)
- Marijuana Withdrawal Checklist – Short Version (15-item)
- Penn Craving Scale (5-item)

Other Drug Use

- Alcohol Use Disorders Identification Test – Self Report (10-item)
- Fagerstrom Test for Nicotine Dependence (6-item)

Psychopathology (depressive symptoms, psychotic symptoms, anxiety)

- Quick Inventory of Depressive Symptomology (16-item)
- State Trait Anxiety Inventory (20-item)

Wellbeing and Quality of Life

- Quality of Life and Satisfaction Questionnaire – Short Form (16-item)
- Pittsburgh Sleep Quality Index (24-item)
- Conor Davidson Resilience Scale (25-item)
- Apathy Evaluation Scale – Self Report (18-item)
- Warwick Edinburgh Mental Wellbeing Scale (14-item)

Weekly Qualtrics Questionnaire via Smart Phone

Participants will be sent the Qualtrics link to their email and will be asked to complete the survey within 24 hours.

- Timeline Follow Back Procedure to measure weekly cannabis use
- Adverse Events
- Patient Health Questionnaire (9-item)
- State Anxiety Inventory – Short Form (6-item)
- Warwick Edinburgh Mental Wellbeing Scale – Short Form (7-item)
- Severity of Dependence Scale (5-item)
- Marijuana Withdrawal Checklist – Short Version (15-item)
- Penn Craving Scale (5-item)
- Visual Analogue Mood Scale: 100-point sliding scale indicating how the intensity of the following feelings over the last week:
  - Mood: happy – sad
  - Satisfaction: content – discontent
  - Cannabis craving: no craving – high craving, never – always
  - Energy: energized – lethargic

Weekly Call

- Identify and workshop barriers to engaging in EXE
- Check-in that EXE and weekly questionnaire has been completed
- Plan for week ahead (offer training with researcher, goal setting etc.)

Cognitive

Rey Auditory Verbal Learning Test (RAVLT): The RAVLT is a hand-administered task that evaluates a wide diversity of functions: short-term auditory-verbal memory, rate of learning, learning strategies, retroactive, and proactive interference, presence of confabulation of confusion in memory processes, retention of information, and differences between learning and retrieval. Participants are given a list of 15 unrelated words repeated over five different trials and are asked to repeat. Another list of 15 unrelated words are given and the client must again repeat the original list of 15 words and then again after 30 minutes.

Paired Associates Learning (PAL) Task: The computer-administered PAL task will be used to measure visual memory. Boxes are displayed on the screen and are "opened" in a randomised order. One or more of them will contain a pattern. The patterns are then displayed in the middle of the screen, one at a time and the participant must select the box in which the pattern was originally located. If the participant makes an error, the boxes are opened in sequence again to remind the participant of the locations of the patterns.

Cardiopulmonary exercise testing (CPET)

All participants will undergo two cardiopulmonary exercise testing (CPET) sessions to obtain a VO_2 peak, heart rate peak, ventilation rates and respiratory exchange ratios. The assessment itself will take approximately 10 minutes, however participants will be asked to allow 45 minutes to accommodate pre-test and post-test procedures. They will perform a graded

exercise test to volitional exhaustion on a treadmill. The starting metabolic intensity (METS) will be 4.6, increasing 2.7 METS every 3 mins (i.e. starting speed at 3kph, increasing 2kph every 3 mins; and incline is fixed at 9% gradient). End-test criteria is determined by participant's subjective indication to stop the test, or for any safety reason determined by the supervising Exercise Physiologist. During the CPET, inhaled and expired gas will be continually analysed every 10 secs using an on-line breath-by-breath system (ADInstruments). Ratings of perceived exertion (Borg Scale) will be recorded every 60 secs. Participants will wear a face mask during the CPET to allow ADInstruments' hardware and software to capture inhaled and expired gases. All CPET sessions will be conducted at BrainPark.

Heart Rate

Each participant will wear a Polar heart rate strap during exercise sessions, and Polar software will be used for data collection. The Polar hardware and software allows real time monitoring and post-session raw data analysis via an online portal. Heart rate monitoring aids the supervising exercise physiologists in making necessary modifications to participant exercise prescription, and verbal feedback, to ensure heart rate responses are reasonably achieving protocol targets. At the completion of data collection the research team will use the final data set to analyse volumes of time spent above key heart rate thresholds - allowing between-, and within-, group comparisons.

Engagement

The research team will document participant attendance for each exercise session to quantify individual, and group level, engagement rates.

MRI

Participants will complete a 1hr MRI session on a 3T Siemens Skyra located on-site at Monash Biomedical Imaging building. Scanning will involve T1-weighted, and T2-weighted image sequence for high-resolution images; diffusion-weighted sequence (DTI) to probe the microstructural content of brain white matter. This sequence will consist of 60-diffusion-encoding gradients (b=3000 s/mm^2; 2.5 mm^3 voxel size).

The scanning sequence is as follows:

1. Structural scan 1: T1-weighted (5.5min)
2. Structural scan 2: T2-weighted (5min)
3. Left Hippocampal MRS (12min)
4. Resting state fMRI (8min)
5. DTI (12 min)
6. fMRI with figural memory cognitive task (7min)

Figural memory task

The figural memory task (Jamadar et al., 2013) is a visual encoding and recognition task designed to minimize verbal encoding of picture stimuli. The task stimuli (20 targets and 20 distractors) consisted of black line drawings presented against a white background.

Participants perform an encoding phase and a recognition phase during fMRI scanning. During the encoding phase 20 target stimuli are presented (duration 3 s, interstimulus interval (ISI) 4 s), which participants were instructed to memorise. Participants press a response box button following each stimulus presentation to confirm that they saw the stimulus. The recognition phase follows the encoding phase after a 12-min delay (with no other cognitive task presented during the delay). During the recognition phase, 20 target and 20 distractor stimuli are presented in a fixed pseudo-random order, each for 3 s with an ISI of 4 s. Participants held the button box in their non-dominant hand and pressed a button with the index finger of their dominant hand. The button on the right (“old”) or button on the left (“new”) fingers to indicate whether they had previously seen each stimulus, and accuracy was emphasized over speed.

Biological Assays

Urine

Urine samples will be collected at baseline and end of intervention to measure the presence of THC-COOH, a secondary metabolite of THC present in cannabis. Prior to collection, participants will be asked to wash their hands to eliminate any possible adulterating or contaminating substances from under participant's fingernails. Participants will be provided with a clean, unused urine specimen collection container and requested to fill the container at least half full (a minimum of 30 mL's) with their urine. Participants will be provided with a private toilet stall for the procedure. Urine specimens will be stored in a secure deep-freezer until the specimen is ready to be shipped to a toxicology laboratory for analysis.

Hair

Hair samples will be collected at baseline and at the end of intervention assessments to measure the presence of THC and THC-COOH using laboratory-recommended procedures, whereby collection will be performed within a secure contamination free room with access restrictions in place. The collector will wear gloves and use clean tools to avoid any risk of interindividual contamination. The posterior vertex region of the head will be the preferred sampling site as this region is associated with the smallest variation in growth rate. A lock of hair that is approximately 200mg will be tied, cut as close to the skin as possible and collected. The root end of scalp hairs will be aligned and clearly identified. The sample will be firmly secured, e.g. with an aluminum foil, to maintain integrity and avoid contamination. Hair samples will be stored in a dry, dark environment at room temperature until the specimen is ready to be shipped to a toxicology laboratory for analysis.

Saliva

Participants will be asked to provide a saliva sample at baseline to measure candidate genes that may influence individuals’ response to the EXE intervention. Samples will be collected utilising Oragene saliva collection kits. Oragene tubes will be labeled prior to sample collection with participant ID and date. The participant will be asked not to eat, drink (including water), smoke or chew gum for 30 minutes before giving the saliva sample. While Wearing Gloves the researcher will hand the assembled oragene tube to the participant and

ask them to spit into the funnel of the tube until the amount of liquid (excluding bubbles) reaches the fill line this should take approximately five minutes. The researcher will then ask the participant to close the funnel lid and then bend the lid over the catchment area until it snaps closed and the liquid is released into the collection tube. The tube will be wiped down with ethanol to remove traces of saliva from the tube. The samples will be stored in the MBI biohazard room with restricted access. Saliva samples will be kept until all samples are collected from the cohort prior to being sent for analysis. The saliva samples can be stored for 5 years at room temperature. Saliva samples will be sent to the Australian Genome Research Facility where they will undergo a DNA extraction and replication process before SNP genotyping using Agena Bioscience MassARRAY mass spectrometry. A candidate genes approach will be employed to assess individual differences in the response to the intervention. Candidate genes will be selected based on priori knowledge regarding genes which are known to underpin the neurobiological processes which may influence response to the intervention.

Assessment of Adverse Effects

Participants will receive weekly telephone calls to monitor for adverse events associated with physical exercise and general study protocol. Adverse events will also be monitored in session by the exercise physiologists.

Payment

Participants will receive $15 compensation for each exercise training sessions and $50 compensation for each of the three clinical assessments attended (baseline, 12-weekss and 6-months). These monies will be provided to compensate participants for the substantial time and effort involved in attending 12-weeks of frequent exercise and assessment sessions and contribute to parking / transport costs.

Recruitment

Large-scale community advertising via print media will be used to advertise the study. Flyers will be placed throughout local health centres, medical centres, community centres, community notice boards, university campuses, on the internet and social media. The study will also be advertised via Directline, a 24/7 alcohol and drug helpline run by Turning Point.

Motivational Strategies *(applied equally to both EXE conditions)*

All trial staff will be trained in person centred health behaviour change methodology (Health Change Associates Ltd). During baseline assessment, participants' knowledge about the health benefits of exercise, the importance of achieving these outcomes, and their readiness and confidence to engage in the intervention will be evaluated and addressed. Potential behavioural, emotional, situational, and cognitive barriers and facilitators to engagement will also be identified. Throughout the trial, Research Officers and AEPs will use this information to guide the following supports.

Calls: Weekly check-ins to reinforce positive performance and workshop barriers preventing engagement for physical exercise. Also monitor adverse events, and help plan and schedule the upcoming week with the participant.

Psychoeducation:
Impact of exercise on brain, physical health and wellbeing emphasised in screen call to promote initial engagement. These themes will be discussed in more detail during consent and emphasised consistently throughout trial participation.

Motivational interviewing: Where appropriate, MI strategies used to assist participants to self-generate strategies to overcome internal or external barriers to engagement.

Planning: Planning for exercise schedule and advance preparation of solutions are common barriers to participation. At the end of their participation a brief report summarising their outcome measures (excluding MRI data) will be provided to them.

Exercise with a researcher: research team will attend EXE classes where feasible to build rapport and help engage participants in training programs.

Statistical analysis plan

Primary Outcome: Hippocampal integrity will be analysed using a linear regression model with a clustered sandwich estimator to account for the repeated measures and will include the main effects of intervention group (EXE [+/–] and time [0-, 3-months]), as well as their interaction (see Fig. 5), whilst adjusting for potential confounders (to be specified in the SAP). For Hypothesis 1, greater efficacy of EXE+ will be confirmed if there is significantly greater change in hippocampal integrity at 3-months relative to baseline in the EXE+ compared to EXE– group.

Secondary Outcomes: Hypothesis 2 will be tested using linear mixed models with restricted maximum likelihood estimation (REML) for handling missing values. Particularly, the models will compare changes in outcomes from 'baseline to 3- months' and 'baseline to 6-months', with main effects of intervention group (EXE [+/–]) and time plus their interaction. The benefits of EXE+ will be supported if there is significantly greater change in the behavioural and functional outcomes measured at 3-months, and/or at 6-months (relative to baseline), in the EXE+ compared to EXE– group. Hypothesis 2b will test whether the changes in behavioural (cannabis use) and functional (cognitive, mental health, general well-being) outcomes (i.e., Hypothesis 2a) are mediated by the changes in hippocampal integrity (i.e., Hypothesis 1). We will test this prediction using longitudinal mediation models [43].

References

1. AIHW, *2010 National Drug Strategy Household Survey*, in *Report 25; PHE 145*, D.S. Series, Editor. 2011: AIHW, Canberra.
2. Moore, T.H., et al., *Cannabis use and risk of psychotic or affective mental health outcomes: A systematic review.* Lancet, 2007. **370**(9584): p. 319-28.
3. Solowij, N., et al., *Cognitive functioning of long-term heavy cannabis users seeking treatment.* JAMA, 2002. **287**(9): p. 1123-31.
4. Solowij, N., et al., *Verbal learning and memory in adolescent cannabis users, alcohol users and non-users.* Psychopharmacology (Berl), 2011. **216**(1): p. 131-44.
5. Hall, W. and L. Degenhardt, *Adverse health effects of non-medical cannabis use.* Lancet, 2009. **374**(9698): p. 1383-91.
6. Broyd, S.J., et al., *Acute and Chronic Effects of Cannabinoids on Human Cognition-A Systematic Review.* Biol Psychiatry, 2016. **79**(7): p. 557-67.
7. Vandrey, R. and M. Haney, *Pharmacotherapy for cannabis dependence: how close are we?* CNS Drugs, 2009. **23**(7): p. 543-53.
8. Gates, P., *et al., Barriers and facilitators to cannabis treatment.* NCPIC Tech.Rep. #1, 2009.
9. Yucel, M., et al., *White-matter abnormalities in adolescents with long-term inhalant and cannabis use: a diffusion MRI study.* J Psychiatry Neurosci, 2010. **35**(6): p. 409-12.
10. Cheetham, A., et al., *Orbitofrontal volumes in early adolescence predict initiation of cannabis use: a 4-year longitudinal study.* Biol Psychiatry, 2012. **71**(8): p. 684-92.
11. Solowij, N., et al., *Alteration to hippocampal shape in cannabis users with and without schizophrenia.* Schizophr Res, 2013. **143**(1): p. 179-84.
12. Zalesky, A., et al., *Effect of long-term cannabis use on axonal fibre connectivity.* Brain, 2012. **135**(Pt 7): p. 2245-55.
13. Yucel, M., et al., *Regional brain abnormalities associated with long-term heavy cannabis use.* Arch Gen Psychiatry, 2008. **65**(6): p. 694-701.
14. Yucel, M., et al., *Hippocampal harms, protection and recovery following regular cannabis use.* Transl Psychiatry, 2016. **6**: p. e710.
15. Cousijn, J., et al., *Grey matter alterations associated with cannabis use: Results of a VBM study in heavy cannabis users and healthy controls.* Neuroimage, 2012. **59**(4): p. 3845-51.
16. Ashtari, M., et al., *Medial temporal structures and memory functions in adolescents with heavy cannabis use.* J Psychiatr Res, 2011. **45**(8): p. 1055-66.
17. Demirakca, T., et al., *Diminished gray matter in the hippocampus of cannabis users: possible protective effects of cannabidiol.* Drug Alcohol Depend, 2011. **114**(2-3): p. 242-5.
18. Johnston, J., et al., *Lithium carbonate in the management of cannabis withdrawal: an RCT in an inpatient setting.* Psychopharmacology (Berl), 2014. **231**(24): p. 4623-36.
19. Volkow, N., et al., *Adverse health effects of marijuana.* NEJM, 2014. **370**(23): p. 2219-27.

20. Erickson, K.I., et al., *Exercise training increases size of hippocampus and improves memory.* Proc Natl Acad Sci U S A, 2011. **108**(7): p. 3017-22.
21. Erickson, K.I., et al., *Brain-derived neurotrophic factor is associated with age-related decline in hippocampal volume.* J Neurosci, 2010. **30**(15): p. 5368-75.
22. Thomas, A.G., et al., *Multi-modal characterization of rapid anterior hippocampal volume increase associated with aerobic exercise.* Neuroimage, 2015. **131**: p. 162-170.
23. Pajonk, F.G., et al., *Hippocampal plasticity in response to exercise in schizophrenia.* Arch Gen Psychiatry, 2010. **67**(2): p. 133-43.
24. Johansen-Berg, H. and E. Duzel, *Neuroplasticity: Effects of Physical and Cognitive activity on brain structure and function.* Neuroimage, 2016. **131**: p. 1-3.
25. Gates, P.J., et al., *Randomized controlled trial of a novel cannabis use intervention delivered by telephone.* Addiction, 2012. **107**(12): p. 2149-58.
26. Suzuki, A., et al., *Astrocyte-neuron lactate transport is required for long-term memory formation.* Cell, 2011. **144**(5): p. 810-23.
27. Ferris, L.T., et al., *The effect of acute exercise on serum brain-derived neurotrophic factor levels and cognitive function.* Med Sci Sports Exerc, 2007. **39**(4): p. 728-34.
28. Park, H. and M.M. Poo, *Neurotrophin regulation of neural circuit development and function.* Nat Rev Neurosci, 2013. **14**(1): p. 7-23.
29. Brown, R.A., et al., *A Pilot Study of Aerobic Exercise as an Adjunctive Treatment for Drug Dependence.* Ment Health Phys Act, 2010. **3**(1): p. 27-34.
30. Buchowski, M.S., et al., *Aerobic exercise training reduces cannabis craving and use in non-treatment seeking cannabis-dependent adults.* PLoS One, 2011. **6**(3): p. e17465.
31. Roessler, K., *Exercise treatment for drug abuse.* Scand J Pub Health, 2010. **38**(6): p. 664-9.
