# Supplementary material for: High-Intensity Exercise and Hippocampal Integrity in Adults With Cannabis Use Disorder: A Randomized Clinical Trial
Source: JAMA Psychiatry. 2025 Sep 10;82(12):1240–5. doi: 10.1001/jamapsychiatry.2025.2319 (PMC12423954; doi:10.1001/jamapsychiatry.2025.2319)
Supplement: Supplement 4. — Data sharing statement [file jamapsychiatry-e252319-s004.pdf]

Data Sharing Statement

Richardson. High-Intensity Exercise and Hippocampal Integrity in Adults With Cannabis Use Disorder. *JAMA Psychiatry*. Published September 10, 2025.
doi:10.1001/jamapsychiatry.2025.2319

Data
Additional Information: NCT04902092
Data available: No

Additional Information
Explanation for why data not available: The data used in this study cannot be made publicly available due to privacy reasons. Participants did not consent to sharing data with a third party for future research. Readers may contact Professor Murat Yucel (Murat.Yucel@qimrberghofer.edu.au) regarding the data.
